# The impact of the COVID-19 pandemic on suicide rates in Hungary: an interrupted time-series analysis

**DOI:** 10.1186/s12888-022-04322-2

**Published:** 2022-12-09

**Authors:** Tamás Lantos, Tibor András Nyári

**Affiliations:** Department of Medical Physics and Informatics, Faculty of Medicine, University of Szeged, 9 Korányi alley, 6720 Szeged, Hungary

**Keywords:** COVID-19, Suicide, Hungary, Trends, Regression, Interrupted time-series, Gender, Age group, Region, Educational attainment

## Abstract

**Background:**

From 2010 to 2019, suicide mortality fell steadily and substantially in Hungary: the declining trend remained stable, and the suicide rate decreased by more than one-third which was remarkable even from an international perspective. However, despite the declining trend, regional inequalities have always characterised the distribution of suicide mortality in Hungary. Following these favourable trends, COVID-19 appeared in Hungary on the 4^th^ of March 2020 which might lead to an increase in suicides. We aimed to investigate this hypothesis in Hungary by gender, age, educational attainment, and region, as well.

**Methods:**

To test whether the pandemic changed the declining trend of Hungarian suicide rates, the observed number of suicides during March–December 2020 (pre-vaccination period) was compared with the expected numbers (without the appearance of COVID-19). An interrupted time-series analysis was conducted by negative binomial regression using monthly data from January 2010 to February 2020 (pre-pandemic period).

**Results:**

Suicide mortality increased significantly compared to the trend during the pre-pandemic period: overall (by 16.7%), among males (18.5%), in the age group 35–49 years (32.8%), and among vocational school graduates (26.1%). Additionally, significant growths in suicide rates were detected in the two regions (Central Hungary and Central Transdanubia) with the lowest COVID mortality rates (by 27.3% and 22.2%, respectively).

**Conclusions:**

Our study revealed reversed trend in suicide mortality during the pre-vaccination period compared to the pre-pandemic period in Hungary. There were significant differences in the pattern of suicide rates by gender, age group, educational attainment, and region during the pre-vaccination period in Hungary, which might be attributed to the socio-economic effects of the COVID-19 pandemic. These findings could prove useful in preventive strategies as the identification of groups at higher risk may be important for suicide prevention; however, further investigations are needed to explore the reasons.

**Supplementary Information:**

The online version contains supplementary material available at 10.1186/s12888-022-04322-2.

## Background

From 2000 to 2019, suicide deaths nearly halved; however, after 2006, the decline of the suicide rate was broken and stagnated for several years [1]. After 2010, suicide mortality fell steadily and substantially again: the declining trend remained stable, and the suicide rate decreased by more than one-third which was remarkable even from an international perspective.

Following these favourable trends, the COVID-19 outbreak appeared at the end of 2019 in Wuhan, Central China. On 11 March 2020, the World Health Organisation (WHO) declared the outbreak a pandemic. The first case and first death in Hungary were officially registered in early- and mid-March 2020, respectively.

In addition to the damaging effects of COVID-19 on the central nervous system [2], there are some social effects of the pandemic that increase the stress levels of individuals [3]: isolation, entrapment, disruption of normal routine, interpersonal conflicts, fear of infection, unemployment. The increased workload in the whole health care system impairs access to mental health services for chronic psychiatric patients, which can also lead to worsening of their condition.

In Hungary, the COVID-19 pandemic increased the number of excess deaths. Similarly, during the COVID-19 epidemic (between March and December 2020), there were almost 11% more suicides reported in Hungary (1438) than in the same period the year before (1294 suicides) [4]. Additionally, the number of male suicide deaths increased to an even greater extent (by more than 14%; from 961 to 1100) during the same period in Hungary.

The effect of the COVID-19 pandemic on suicide mortality by gender was examined in detail in a recent study [5]. However, regional inequalities have always characterised the distribution of suicide mortality in Hungary. We might hypothesise that the excess mortality could be also observed in suicide mortality (for certain subpopulations) compared to the 10 years before the pandemic. Our aim was to investigate this hypothesis in Hungary by gender, age, educational attainment, and region, as well.

## Methods

### Study population and suicide data

Data on the population were obtained from the published nationwide population register operated by the *Hungarian Central Statistical Office* (HCSO; [4]). The annual mid-year population estimates were used since there were no monthly population data available.

As trend stability in the (pre-)period is crucial in interrupted-time series, the 10 years between 2010 and 2019 were considered in this analysis since the annual trend in suicide rates for the whole population remained stable during this period, unlike in previous years. The data on (monthly) suicide deaths were also available on the online HCSO database [4]. These data were classified according to the International Classification of Diseases, 10^th^ revision (ICD-10); codes concerning “intentional self-harm” were X60-X84 and Y87.

Suicide rates (SRs) were expressed per 100,000 population per year using the annual mid-year population estimates for the relevant year. The suicide rates were directly standardised by age [6] using the *Revised European Standard Population* (RESP) published in 2013 [7] to facilitate a comparison of rates over time by removing the effect of age composition.

Population and suicide deaths were initially broken down by *age group* as follows: 0–19 ("youth"),

20–34 ("young adults"), 35–49 ("middle-aged adults"), 50–64 ("older adults") and over 65 years ("elderly"/pensioners). The distribution of the RESP was as follows: 0–19 years 21.5%, 20–34 years 18.5%, 35–49 years 21%, 50–64 years 19.5%, and over 65 years 19.5%. Nonetheless, due to the lower number of cases in the under-20 age group, the first two groups were analysed together.

**Fig. 1:**
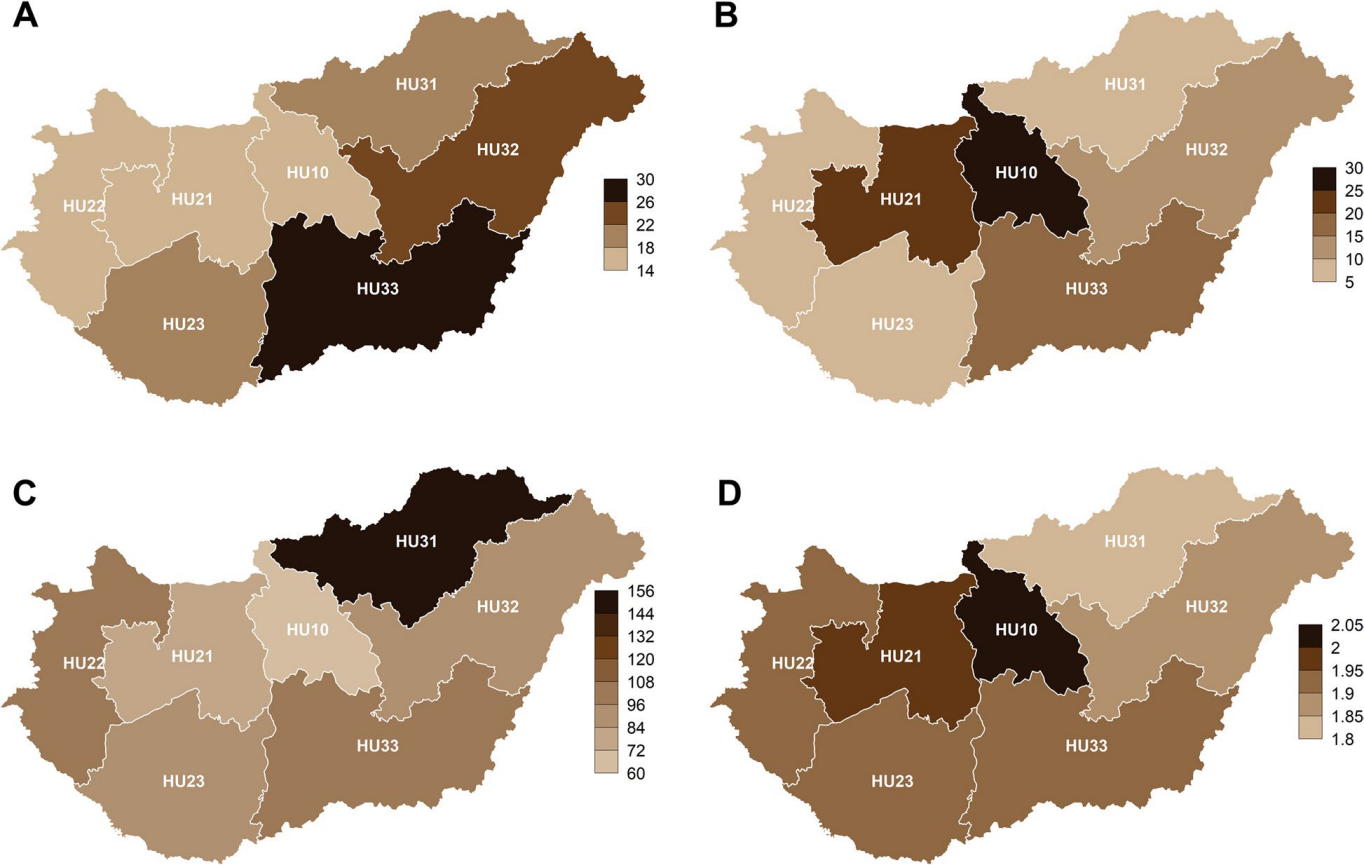
NUTS2 regions of Hungary. Regions were coloured by: **A.** Suicide mortality rates during 2010–2019 **B.** IRRs for suicide deaths during the pre-vaccination period **C.** COVID-19 death rates during the pre-vaccination period **D.** Divorce rates during 2012–2019. Incidence rate ratios (IRRs) were obtained from negative binomial (NB) regression and expressed as percentage increases with respect to the expected numbers that would have occurred without the appearance of COVID-19. COVID-19 death rates and divorce rates were expressed per 100,000 capita and per thousand capita, respectively. The maps depicted in the figure are the authors’ own work and were created by using packages *rgdal* (v1.5-32; https://cran.r-project.org/web/packages/rgdal/index.html) and *cartography* (v3.0.1; https://cran.r-project.org/web/packages/cartography/index.html) in R. Notes: *NUTS2* Nomenclature of territorial units for statistics (from the French version *Nomenclature des Unités territoriales statistiques*), 2^nd^ level; Codes: *HU10* Central Hungary, *HU21* Central Transdanubia, *HU22* Western Transdanubia, *HU23* Southern Transdanubia, *HU31* Northern Hungary, *HU32* Northern Great Plain, *HU33* Southern Great Plain. On January 1, 2018, Hungary split the region of Central Hungary into two new regions [Government Decision No. 2013/2015 (XII. 29.)]: the Budapest region (containing the capital of the same name; previously *HU102*) and the surrounding region of Pest (previously *HU101*). In favour of continuity, we examined the territorial units existing before January 1, 2018, and used their names.

The second level of the NUTS 2013 classification (*Nomenclature des unités territoriales statistiques* – Nomenclature of Territorial Units for Statistics, 2013 revision) [8] served as the foundation for the territorial units. The seven *regions* of Hungary were as follows (Fig. 1A): Central Hungary (HU10), Central Transdanubia (HU21), Western Transdanubia (HU22), Southern Transdanubia (HU23), Northern Hungary (HU31), Northern Great Plain (HU32) and Southern Great Plain (HU33).

Educational attainment was categorised according to the ISCED-97 system (International Standard Classification of Education, 1997 version) [9, 10]: less than eight years of primary school (ISCED grades 0-1A), eight years of primary school (2A), vocational qualification without a secondary school-leaving certificate (vocational schools, 2C-3C), secondary school-leaving certificate (secondary vocational schools and grammar schools, 3A) and higher education degree (colleges and universities, 5A). Population data were only available for the group aged 15–74 years; in addition, the first two groups were aggregated as “at most primary school” due to their low number of cases and the “unstable” (i.e. rapidly decreasing by year) population of the first group (“0–7 grades”).

### Statistical analyses

The observed SR during the pre-vaccination period of the COVID-19 pandemic (March − December 2020) was compared with the expected SR based on the pre-COVID-19 period (January 2010 − February 2020). Since the Hungarian SRs decreased in the last years, an interrupted time series (ITS) analysis was conducted to control for annual trends and seasonal variations [11]. In the absence of “intervention” (i.e. the COVID pandemic), this trend would remain “unchanged”. This is a common method to apply in such situations. In the current phase of the analysis, we only investigated the effect of the “intervention”, no other structural indicators were considered.

Accordingly, the following segmented regression impact model (with a level change) was used:

$${Y}_{t}={\beta }_{0}+{\beta }_{1}T+{\beta }_{2}{X}_{t}$$

where ${Y}_{t}$ is the outcome (suicide deaths) at time $t$ (where $t$ is measured in months), $T$ is the time since the beginning of the study (measured in months), ${X}_{t}$ is a dummy variable indicating either the pre-intervention period (“pre-COVID period”, coded 0) or the post-intervention period (“pre-vaccination period”, coded 1). The baseline level at $T=0$ is represented by ${\beta }_{0}$, the change in outcome associated with a time unit (month) increase is represented by ${\beta }_{1}$ (indicating the underlying pre-intervention trend), and the level change after the intervention is represented by ${\beta }_{2}$.

Due to over-dispersion (i.e. variance exceeding the mean), we conducted a negative binomial (NB) regression analysis to identify suicide trends in the pre-COVID-19 period, with the log-transformed (and age-standardised) mid-year population as offset variable (to convert the count into a rate and adjust for population changes over time). Seasonal effects were adjusted by harmonic (Fourier) terms using pairs of sine and cosine functions (in this case two of them):

$$\mathrm{cos}\left(\frac{2k\pi m}{12}\right), \mathrm{sin}\left(\frac{2k\pi m}{12}\right), k=\mathrm{1,2}$$

where $m$ represents the month (for January: $m=1$; for February: $m=2$ etc.).

To test whether SRs during the COVID-19 pandemic were out of line with the declining trend of previous years, expected and observed suicides of the COVID-19 period were compared by calculating incidence rate ratios (IRRs) and 95% confidence intervals (CIs).

Analyses were carried out overall and then separately by sociodemographic factors (gender, age, educational attainment, and region, respectively), as stratified by levels of a single factor. Consequently, the models mentioned above included the corresponding stratum-specific population (broken down by levels of the given factor) as an offset variable.

To ensure the robustness of our results, we performed sensitivity analyses for a shorter period, starting in January 2015 (instead of January 2010).

In favour of completeness, suicide rates during the pre-vaccination period were also compared directly to the same period of the previous year (i.e. March − December 2019).

*p*-values less than 0.05 were considered statistically significant. All analyses were performed using R (v4.1.2; R Core Team 2021).

## Results

Table S1 (see Additional File 1) displays summary statistics for suicide cases and the corresponding population at risk during various periods. The number of population and suicides were expressed as monthly averages to make periods comparable. Percentages were expressed as proportions of the total number (which can be slightly different due to protected data).

During the pre-COVID-19 period between January 2010 and February 2020, the average number of suicide deaths in Hungary was 164 per month. There were 23 fewer suicides per month between January 2015 and February 2020 (which represents the “second half” of the previous period); this indicates a decrease in the monthly suicide rate of approximately 13%. In addition, rates of suicide decreased at each level of each factor.

During the pre-vaccination period of the pandemic (i.e. March − December 2020), 1438 suicides (144 per month on average) were registered in Hungary; this was about 11% more than in the same period of the previous year (1294; 129 monthly). Additionally, suicide rates increased in almost all subgroups: the regions of Western and Southern Transdanubia were the only exceptions.

The interrupted time-series analyses were performed for the whole population and separately by gender, age group, educational attainment, and region, respectively (Table 1).

**Table 1:** Incidence rate ratios (IRRs) for suicide deaths during March-December 2020 using negative binomial (NB) regression

Subgroup	Compared to the expected rates based on the period Jan. 2010 – Feb. 2020					Compared to the expected rates based on the period Jan. 2015 – Feb. 2020				
	IRR	95% CI			*p*-value	IRR	95% CI			*p*-value
*Total*	1.167^a^	1.083	-	1.259	< 0.001	1.123^a^	1.034	-	1.22	0.006
GENDER										
* Male*	1.185^a^	1.089	-	1.288	< 0.001	1.12^a^	1.018	-	1.233	0.02
* Female*	1.105	0.957	-	1.277	0.173	1.126	0.972	-	1.303	0.113
AGE GROUP										
* 0–34 years*	1.103	0.907	-	1.341	0.325	1.127	0.892	-	1.423	0.315
* 35–49 years*	1.328^a^	1.165	-	1.513	< 0.001	1.231^a^	1.055	-	1.437	0.008
* 50–64 years*	1.109	0.982	-	1.252	0.096	1.038	0.902	-	1.194	0.607
* 65* + *years*	1.128	0.998	-	1.275	0.053	1.131	1.055	-	1.272	0.042
EDUCATIONAL ATTAINMENT (15–74 YEARS)										
* Higher Education*	1.198	0.96	-	1.496	0.11	1.375^a^	1.057	-	1.789	0.018
* Secondary Education*	1.044	0.902	-	1.208	0.563	1.053	0.884	-	1.254	0.562
* Vocational School*	1.261^a^	1.11	-	1.433	< 0.001	1.159^a^	1.004	-	1.339	0.044
* At most Primary School*	1.178^a^	1.018	-	1.363	0.028	1.071	0.901	-	1.273	0.439
NUTS2 REGION										
* Central Hungary*	1.273^a^	1.132	-	1.432	< 0.001	1.229^a^	1.07	-	1.412	0.004
* Central Transdanubia*	1.222^a^	1.009	-	1.48	0.04	1.276^a^	1.017	-	1.6	0.035
* Western Transdanubia*	1.054	0.843	-	1.318	0.644	1.045	0.806	-	1.356	0.738
* Southern Transdanubia*	1.052	0.847	-	1.307	0.645	0.964	0.753	-	1.235	0.773
* Northern Hungary*	1.063	0.881	-	1.284	0.523	1.057	0.852	-	1.311	0.616
* Northern Great Plain*	1.146	0.99	-	1.325	0.068	1.103	0.929	-	1.309	0.262
* Southern Great Plain*	1.193	1.026	-	1.388	0.022	1.086	0.919	-	1.283	0.334

^a^ Statistically significant increase

**Fig. 2:**
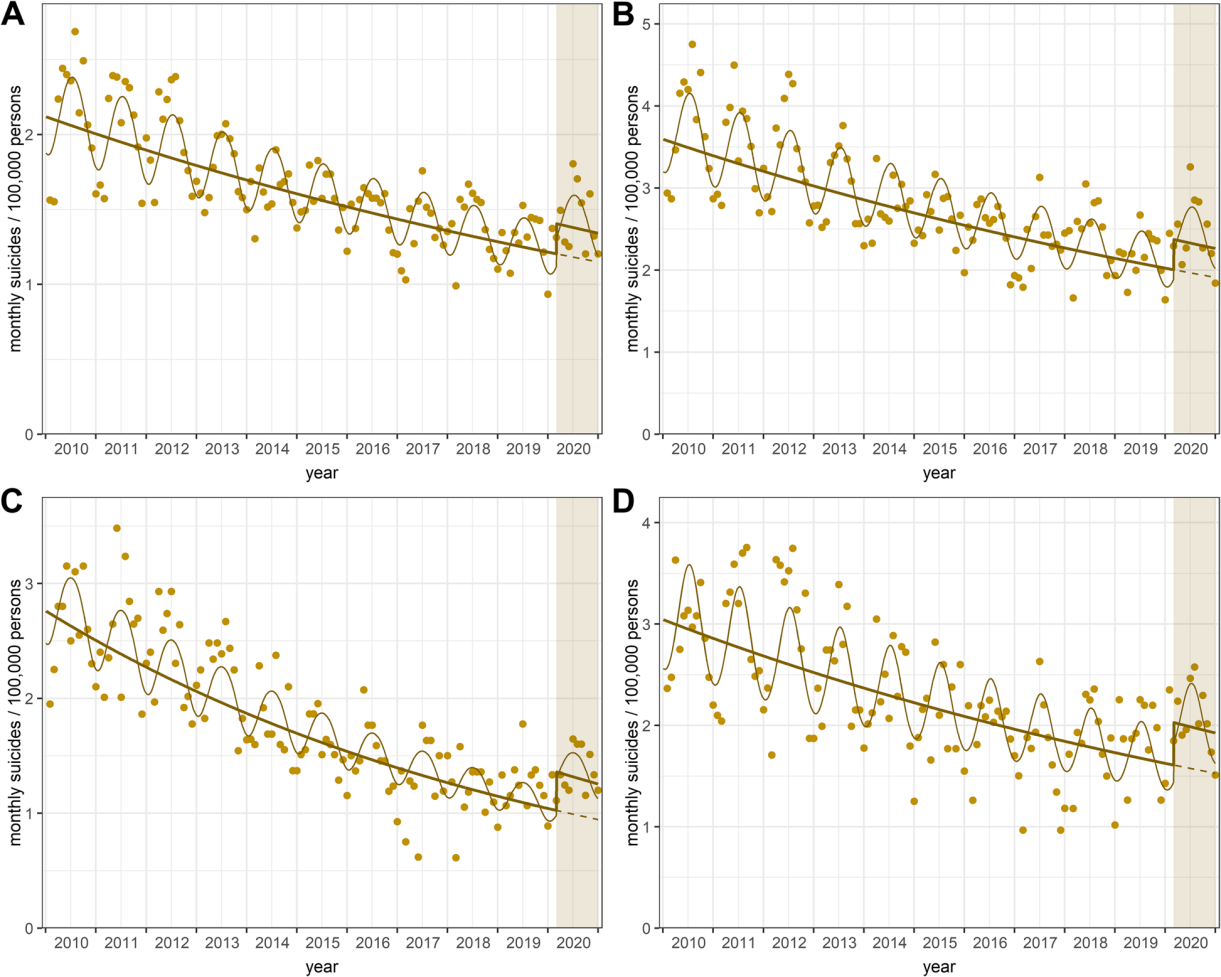
Trends of suicide rates during 2010–2020 for the whole population, males, 35–49-year-olds, and vocational school graduates. Monthly suicide rates per 100,000 persons over time: **A.** Whole population **B.** Males **C.** Age group 35–49 years. **D.** Vocational school graduates. Pre-intervention period: white background; post-intervention period: coloured background; observed rates: points; fitted rates/trends (annual trend and seasonal variation): continuous lines; counterfactual scenario (for annual trend): dashed line. Notes: Only those (sub)groups were displayed here for which the effect of the pandemic on suicide rates was significant both in the main and sensitivity analyses. Regions were displayed on the map (Fig. 1B)

The COVID-19 period showed a significant rise in suicide deaths compared to the projected numbers (in the absence of the COVID-19 pandemic): overall, among men and people aged 35 to 49 (see Fig. 2A-C), respectively. The estimated IRRs were 1.167 (95% CI: 1.083–1.259) for the general population, 1.185 (1.089–1.288) for males, and 1.328 (1.165–1.513) for the group aged 35–49 years, respectively. The results of sensitivity analysis were nearly identical to those previously indicated (except for the age group over 65 years; see Table 1).

Regarding educational attainment, significant increases (compared to the hypothetical trend) in suicide mortality were revealed among those who graduated from vocational school (Fig. 2D) and those who completed at most primary school. The corresponding IRRs were 1.261 (1.11–1.433) and 1.178 (1.018–1.363), respectively. However, in the sensitivity analysis, no significant increase for the latter group was detected (but significant for those with a college or university degree; see Table 1).

There were also differences by region (see Fig. 1B). The increase was significant in the case of.

Central Hungary (HU10), Central Transdanubia (HU21) and the Southern Great Plain (HU33); the corresponding IRRs were 1.273 (95% CI: 1.132–1.432), 1.222 (1.009–1.48) and 1.193 (1.026–1.388), respectively. In the sensitivity analyses, similar significant increases were observed in the regions of Central Hungary and Central Transdanubia but non-significant in the Southern Great Plain (Table 1). Nonetheless, it might also be worth noting that a decrease was only observed in the case of Southern Transdanubia (among the levels of any factors).

**Fig. 3:**
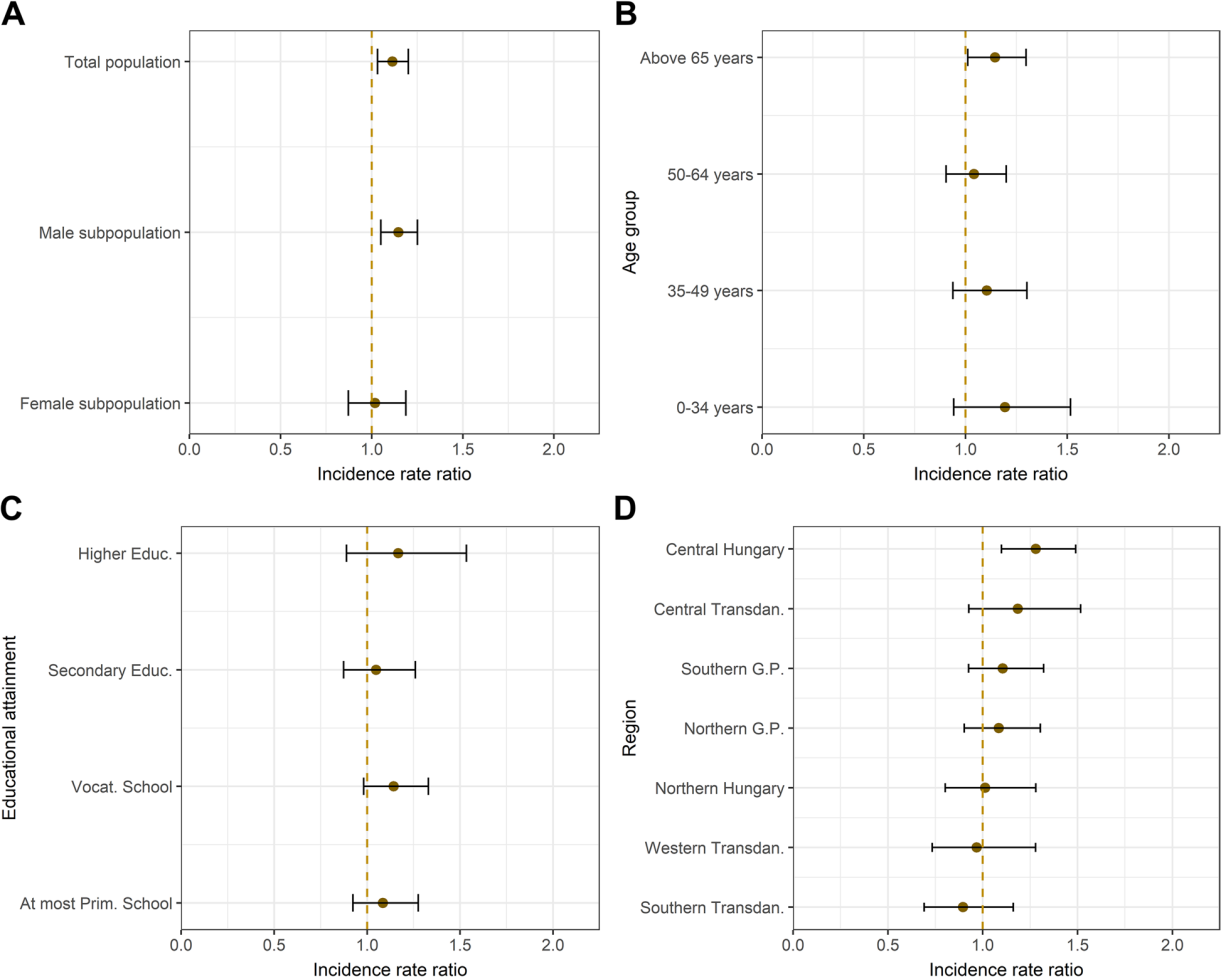
Incidence rate ratios (IRRs) for suicide deaths during March − December 2020 by various sociodemographic factors (compared to the same period of 2019). **A.** Whole population and by gender **B.** By age group **C.** By educational attainment (population aged 15 − 74 years). **D.** By region

Referring to the yearly comparisons, suicide rates increased significantly (compared to the same period of the previous year) overall (IRR = 1.114; 95% CI: 1.032–1.201), among males (1.146; 1.05–1.251), in the age group over 65 years (1.146; 1.011–1.298), and in the region Central Hungary (1.28; 1.099–1.491), respectively (see Fig. 3).

## Discussion

### Main findings

The number of suicides increased significantly by 16.7% compared to the hypothetical trend that would have occurred without the appearance of COVID-19; significant rises were also observed in the male (18.5%) and aged 35–49 years (32.8%) subpopulations; furthermore, within the age group 15–74 years, among vocational school graduates (26.1%). There were also some differences by region: the suicide rate increased significantly only in two regions, in Central Hungary and Central Transdanubia (by 27.3% and 22.2%, respectively).

### Comparison with other studies

Since suicide mortality can increase during infectious disease outbreaks [12], it was hypothesised that the COVID-19 pandemic might also elevate suicide mortality. However, a preliminary international study conducted in April–July 2020 that investigated 21 higher-income countries concluded that there was „showed no evidence of a significant increase in the risk of suicide since the pandemic began in any country or area” [13]. Despite this, it is still unclear how the COVID-19 pandemic has affected suicide deaths or at least can vary by country. Recently published papers have reported static [14], decreasing [15], and even ascending [16] suicide rates in the context of the pandemic.

A recent Hungarian study [5] revealed a significant increase during the first year of the pandemic. One of the possible reasons for this is that social acceptance of suicide is still high, so Hungarian people tend to turn to suicide as a ‘solution’ in a crisis like the pandemic. In addition, ‘normal’ health care had to be cut back due to the pandemic (like everywhere in the world), and psychiatry in Hungary suffered particularly: in many hospitals, beds and specialists were removed from here at the earliest and returned here at the latest.

Gender differences might be explained by social factors (linked to traditional roles; individualism, risk-taking behaviour, independence, economic/employment status). Moreover, although men are generally more exposed and susceptible to social and psychological stress, their compliance with the therapy is poorer than female patients’. Conversely, the increase in suicide mortality (following an initial decline) during the COVID-19 pandemic was more marked for females in Japan [17]. However, it is worth mentioning that from 2010 to 2019, the extent of the decline in suicide rates for men was nearly one and a half times higher than for women in Hungary (41.7% and 29.3%, respectively).

A significant increase in suicide rates was only found in the age group of 35–49 years. This might be attributed to the multiple burdens of being an adult during the pandemic: the members of this age group can be active workers (home office, fear of unemployment), parents (home-schooling, fear of infection), and children of their ageing parents at the same time (care of them, fear of losing them). In contrast to this, a study in Taiwan found that suicide rates decreased in the middle age group [18]. Again, it can be noted that from 2010 to 2019, the suicide rate declined to the largest extent (by 51.8%) in this age group.

Our previous report revealed that among the levels of educational attainment, (by far) the greatest percentage drop in suicide mortality had been seen in the group of people graduating from vocational school and the largest male-to-female ratio had been also observed in this group between 1998 and 2017 in Hungary [19]. Moreover, the strict measures of the first wave temporarily limited the physical presence in the workplace which primarily affected manual workers and craftsmen. Furthermore, in the sectors of accommodation and catering, fishery, and agriculture, the proportion of people who are not able to work from home is over 80 per cent (which could lead to unpaid leave or loss of job) [20]. All the above (including the relatively low base rate) could have contributed to the finding of a significantly increased suicide rate for vocational school graduates during the pre-vaccination period (compared to the hypothetical trend) in Hungary.

Previous studies have reported significantly higher suicide mortality rates in the Northern and Southern Great Plain compared to Central Hungary, which includes the capital city of Hungary (Budapest).

([21, 22]; see Fig. 1A). However, a significant increase in suicide mortality was observed in Central Hungary during the COVID-19 pandemic. This might be caused by the decline in the ‘bustling metropolitan life’ (catering industry, cultural events, tourism) due to the restrictions. In the case of Central Transdanubia, the increase may have similar reasons (because of its relative proximity to Budapest and Lake Balaton), supplemented by the decline in the automotive– and processing industry (on account of factory shutdowns).

Consequently, the regions of Central Hungary and Central Transdanubia were hardest hit by the first wave of COVID-19. At the peak of the first wave, Budapest and the county of Pest (i.e. the region of Central Hungary) were among the most infected counties of Hungary, together with the counties of Fejér and Komárom-Esztergom (parts of the region Central Transdanubia) [23]. Moreover, the registered unemployment rate has significantly increased in Budapest and its agglomeration, along with the settlements around Lake Balaton (partly in the region of Central Transdanubia) [24, 25].

Interestingly, these two regions recorded the lowest mortality rates (directly) due to COVID–19 during the study period in Hungary (Fig. 1C). Additionally, according to the available data, divorce rates were highest in these two regions (Central Hungary and Central Transdanubia) between 2012 and 2019 ([26]; see Fig. 1D).

In summary, our study has described the relationship between the impact of the COVID-19 pandemic and suicide mortality in Hungary during the pre-vaccination period.

### Strengths and limitations

As far as we are aware, this is the first study analysing the suicide trends in Hungary concerning the COVID-19 pandemic by age group, educational attainment, and region. The longest study period (providing the trend stability) was used in the interrupted time-series analyses.

Although the vital statistics performance index of Hungary is one of the best in the world [27] and the percentage of garbage-coded deaths is also quite low in Hungary [28], there can be cause-of-death biases. Moreover, only deaths from “intentional self-harm” were considered in our analyses (this is the usual approach in Hungary even if it might be too restrictive); however, there are other death causes (and ICD-10 codes) which can qualify for deaths by suicide [29]: “poisoning of undetermined intent” (Y10-Y20), “other events of undetermined intent” (Y20-Y34) and “late effects of other events of undetermined intent” (Y87.2). Consequently, the undercounting of suicide deaths (year by year) cannot be ruled out. However, we are confident that our results do reflect real trends.

Some cells in the online HCSO database contained protected data. We considered these ‘missing’ fields to be 1 (as in the vast majority of cases, this must be the true value). This can cause some discrepancies in marginal numbers, but these differences are negligible.

Overdispersion did not influence our results, as the NB regression method was employed in the analyses.

We applied a divided age-group structure and thus calculated age-standardised mortality rates to make suicide rates comparable over time. Although the age variable could have been simply included in our models to control for age composition, we decided to standardise rates instead (as this was the case also in [11]). Notwithstanding the above, age-standardisation did not offer any real advantages (over crude rates) in our analyses as populations at risk were relatively stable over time (and thus, calculations based on non-standardised rates also led to very similar results) in most cases. However, it should be mentioned that age-standardisation was not possible in the case of educational attainment (due to a lack of more detailed data) where the concerning populations were particularly less stable in some cases (especially in the subgroups “0–7 grades” and “higher education”).

The HCSO’s public mortality data have a lead time of two years; that is, there have been no available data for suicide deaths in Hungary during 2021 and 2022 yet. Consequently, we have only been able to investigate suicide deaths till December 2020. Accordingly, as the pandemic progresses, the patterns described here may shift. In addition, it is essential to keep in mind that vaccinations began in Hungary on December 26, 2020; as a result, we can say that the “pre-vaccination period” was analysed. The possibility of ecological fallacy is another limitation of our analysis: individual-level associations may not always be reflected in ecological-level associations.

## Conclusion

Our findings demonstrate how the COVID-19 pandemic affected suicide mortality (significantly increasing suicide rates) in two of Hungary’s most affluent regions before vaccination. Although the underlying causes are unclear, economic variables may help to partially explain this (e.g. shutdowns in the automotive– and processing industry).

To our knowledge, this has been the first study to examine so comprehensively (in terms of socio-demographic factors) the effect of the COVID-19 pandemic on suicide rates in Hungary. In general, there was a significant rise in the number of suicides in Hungary before the vaccination period (compared to the possible trend that would have taken place if the pandemic hadn’t happened). The pattern of suicide related to the COVID-19 pandemic has been described in our study by gender, age group, educational attainment, and region; these patterns were different from one another. Since the identification of groups at higher risk during the pandemic may be crucial to suicide prevention (as the pandemic continues to evolve), these findings may prove useful for preventive strategies. However, further research is required to determine their causes.    

## Supplementary Information

**Additional file 1:** **Table S1.** Monthly averagepopulations and suicide deaths by sociodemographic subgroup.

## Data Availability

The data used in this study are available from the Dissemination Database published online by the Hungarian Central Statistical Office, http://statinfo.ksh.hu/Statinfo/themeSelector.jsp.

## Abbreviations

HCSO: Hungarian Central Statistical Office
ICD: International Classification of Diseases
SR: Suicide rate
RESP: Revised European Standard Population
NUTS: Nomenclature of Territorial Units for Statistics (from the French version Nomenclature des Unités Territoriales Statistiques)
ITS: Interrupted time-series
IRR: Incidence Rate Ratio
CI: Confidence Interval
NB: Negative binomial
