# Supplementary material for: The impact of the COVID-19 pandemic on suicide rates in Hungary: an interrupted time-series analysis
Source: BMC Psychiatry. 2022 Dec 9;22:775. doi: 10.1186/s12888-022-04322-2 (PMC9733003; doi:10.1186/s12888-022-04322-2)
Supplement: Supplementary file 1 — Additional file 1: Table S1. Monthly averagepopulations and suicide deaths by sociodemographic subgroup. [file 12888_2022_4322_MOESM1_ESM.docx]

| Subgroup | Jan 2010 – Feb 2020 | | Jan 2015 – Feb 2020 | | Mar 2019 – Dec 2019 | | Mar 2020 – Dec 2020 | |
| --- | --- | --- | --- | --- | --- | --- | --- | --- |
|  | Population | Suicides | Population | Suicides | Population | Suicides | Population | Suicides |
| *Total* | 9862466 | 164 | 9796790 | 141 | 9771141 | 129 | 9750149 | 144 |
| GENDER | | | | | | | | |
| *Male* | 4701247 (48%) | 125 (76%) | 4679658 (48%) | 106 (75%) | 4678306 (48%) | 96 (74%) | 4672292 (48%) | 110 (76%) |
| *Female* | 5161219 (52%) | 39 (24%) | 5117131 (52%) | 35 (25%) | 5092835 (52%) | 33 (26%) | 5077857 (52%) | 34 (24%) |
| AGE GROUP | | | | | | | | |
| *0-34 years* | 3886028 (39%) | 19 (12%) | 3743832 (38%) | 17 (12%) | 3706317 (38%) | 13 (10%) | 3686172 (38%) | 16 (11%) |
| *35-49 years* | 2214400 (22%) | 39 (24%) | 2268798 (23%) | 31 (22%) | 2248864 (23%) | 28 (22%) | 2229121 (23%) | 31 (22%) |
| *50-64 years* | 1987293 (20%) | 54 (33%) | 1936443 (20%) | 43 (31%) | 1899864 (19%) | 39 (31%) | 1875406 (19%) | 40 (28%) |
| *65+ years* | 1774745 (18%) | 51 (31%) | 1847717 (19%) | 49 (35%) | 1916096 (20%) | 47 (37%) | 1959450 (20%) | 55 (39%) |
| EDUCATIONAL ATTAINMENT (15–74 YEARS) | | | | | | | | |
| *Higher Education* | 1472856 (20%) | 12 (9%) | 1552407 (21%) | 11 (10%) | 1624222 (22%) | 10 (10%) | 1712289 (23%) | 12 (12%) |
| *Secondary Education* | 2404980 (32%) | 28 (22%) | 2427796 (32%) | 26 (24%) | 2428952 (33%) | 24 (24%) | 2414668 (33%) | 25 (23%) |
| *Vocational School* | 1830426 (24%) | 42 (33%) | 1833313 (25%) | 35 (33%) | 1819316 (25%) | 33 (34%) | 1785743 (24%) | 37 (35%) |
| *At most Primary School* | 1838215 (24%) | 45 (36%) | 1655865 (22%) | 34 (32%) | 1546539 (21%) | 30 (31%) | 1496026 (20%) | 32 (30%) |
| NUTS2 REGION | | | | | | | | |
| *Central Hungary* | 2988210 (30%) | 41 (25%) | 3011430 (31%) | 36 (26%) | 3039239 (31%) | 31 (24%) | 3040478 (31%) | 39 (28%) |
| *Central Transdanubia* | 1069571 (11%) | 16 (10%) | 1058590 (11%) | 14 (10%) | 1059496 (11%) | 12 (10%) | 1060171 (11%) | 15 (10%) |
| *Western Transdanubia* | 987615 (10%) | 12 (7%) | 986550 (10%) | 11 (8%) | 991945 (10%) | 11 (8%) | 995712 (10%) | 11 (7%) |
| *Southern Transdanubia* | 909109 (9%) | 14 (9%) | 890100 (9%) | 12 (9%) | 877085 (9%) | 13 (10%) | 872839 (9%) | 12 (8%) |
| *Northern Hungary* | 1163234 (12%) | 20 (12%) | 1139326 (12%) | 17 (12%) | 1122469 (11%) | 15 (12%) | 1115420 (11%) | 15 (11%) |
| *Northern Great Plain* | 1473867 (15%) | 30 (18%) | 1462149 (15%) | 25 (18%) | 1446810 (15%) | 23 (18%) | 1438896 (15%) | 25 (17%) |
| *Southern Great Plain* | 1270860 (13%) | 29 (18%) | 1248645 (13%) | 25 (18%) | 1234097 (13%) | 24 (19%) | 1226633 (13%) | 27 (19%) |

**Table S1.** Monthly average populations and suicide deaths by sociodemographic subgroup.

Note: Percentages do not always add up to 100 due to rounding.
